# Supplementary material for: A Modified Variant of Fasciola hepatica FhSAP-2 (mFhSAP-2) as a Recombinant Vaccine Candidate Induces High-Avidity IgG2c Antibodies and Enhances T Cell Activation in C57BL/6 Mice
Source: Vaccines (Basel). 2025 May 20;13(5):545. doi: 10.3390/vaccines13050545 (PMC12115747; doi:10.3390/vaccines13050545)
Supplement: Supplementary file 1 [file vaccines-13-00545-s001.zip › vaccines-3623219-supplementary.pdf]

Data S1: Detailed Protocol for the Expression and Purification of mFhSAP-2

1. A single colony of transformed *E. coli*-BL21 was inoculated into 5 mL of Luria Broth (LB) medium containing 100 μg/mL kanamycin and grown overnight at 37°C with shaking at 200 rpm.
2. The pre-culture was then transferred to 1 L of LB-kanamycin pH 7.2 and incubated on a continuous shaker (250 rpm) at 37°C.
3. When the absorbance at 600 nm reach 0.6-0.8, the fusion-protein expression is induced by adding isopropylthio-β-galactoside (IPTG) to a final concentration of 0.2mM followed by incubation at 37°C for 4h.
4. After incubation, the cells were harvested by centrifugation at 10,000 x g for 20 min at 4°C.
5. The bacterial pellets (Inclusion bodies) are suspended in denaturing buffers (6M urea, 2M guanidine thiocyanate, 20 mM Tris-HCl, 500 mM NaCl, and 15 mM imidazole) and then sonicated at maximal intensity on ice.
6. The bacterial lysate is centrifuged at 10,000 x g for 20 min at 4°C, then filtered through a 0.45 μm membrane.
7. Lysate is loaded onto a HiTrap Chelating HP column (Cytiva, Malborough, MA, USA) for purification.
8. After elution, the purified mFhSAP-2 is desalted against PBS using a PD-10 column (Cytiva, Malborough, MA, USA).
9. The 6Xhis-GST-mFhSAP-2 is again loaded on to the Ni-colum to be digested with TEV protease and mFhSAP-2 free of tag is then eluted with PBS.
10. mFhSAP-2 is then subgested to sucessive endotoxin removal cycles using polymyxin B (PMB) columns according to the manufacturer's instructions.
11. Once the endotoxin levels assessed using a Chromogenic Limulus Amebocyte Lysate (Lonza, Walkersville, MD), is bellow 0.19 EU/mg, mFhSAP-2 is concentrated by AMICON Ultracentrifugal Filters (YM-3) and its concentration adjusted to ~1.0 mg/mL, as determine by the BCA method.
12. The purified endotoxin-free mFhSAP-2 is stored in aliquots at -80°C until use.

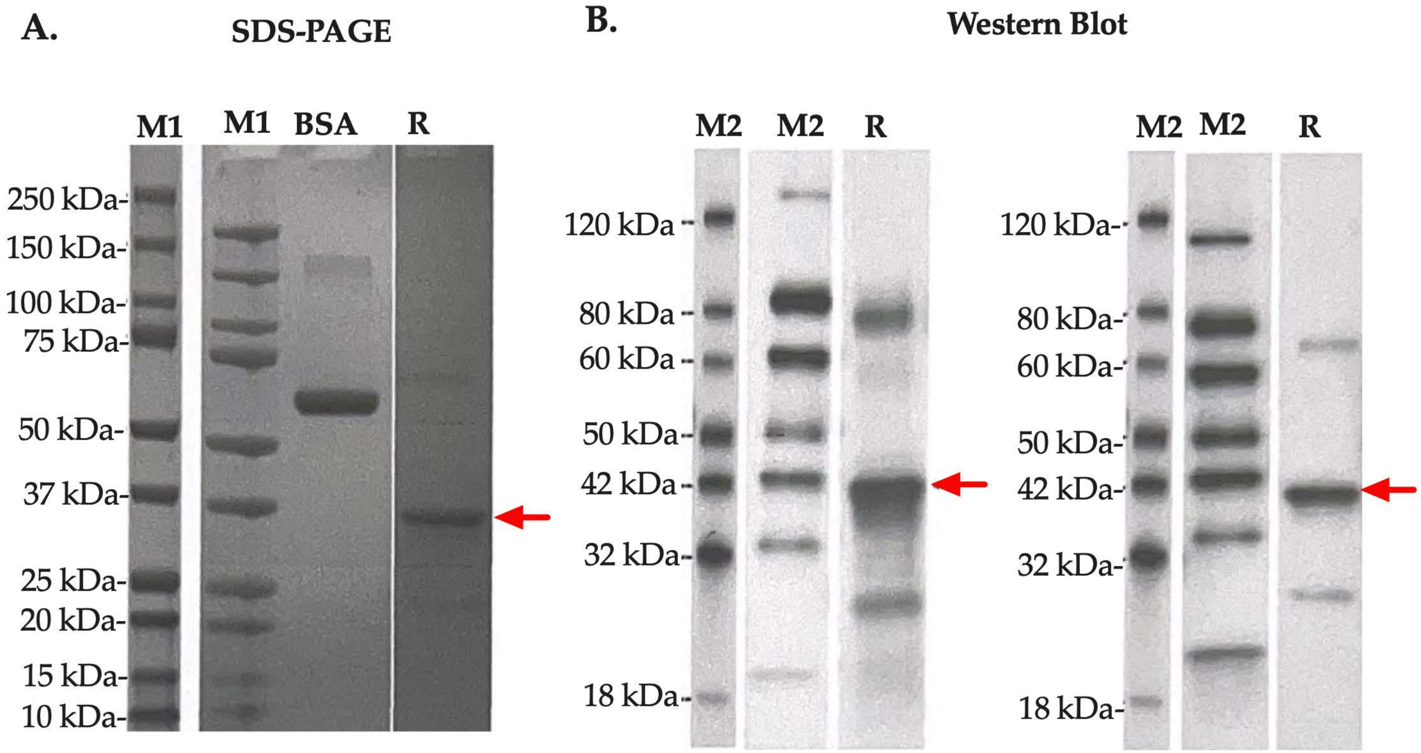

Lane M1: Protein Marker, Bio-Rad Cat. No. 1610374S
Lane M2: Protein Marker, Genscript Cat No. M00521
BSA: 2.00 μg
R: Reducing condition
Primary antibody: Mouse anti-His mAb (Genscript, Cat. No. A00186)
Primary antibody: Rabbit anti-GST, Cat. No. A00097)

Fig. S1. SDS-PAGE and Western Blot analysis of purified mFhSAP-2 fusion protein. The mFhSAP-2 fusion protein (6His-GST-mFhSAP2) was purified by affinity chromatography using a Ni+-column and its purity was evaluated by **(A)** SDS-PAGE stained with coomasie blue and **(B)** Western blot using specific antibodies either against 6His or GST-tags. Red arrow indicate the polypeptide band of ~38.7 kDa corresponding to the mFhSAP-2 fusion protein. SDS-PAGE and Western blot were done and provided by GenScript (Order U091CFK120).
